# Acute oral intake of a higenamine-based dietary supplement increases circulating free fatty acids and energy expenditure in human subjects

**DOI:** 10.1186/1476-511X-12-148

**Published:** 2013-10-21

**Authors:** Sang-Rok Lee, JohnHenry M Schriefer, Trint A Gunnels, Innocence C Harvey, Richard J Bloomer

**Affiliations:** 1 Department of Health and Sport Sciences, Cardiorespiratory/Metabolic Laboratory, University of Memphis, 106 Roane Field House, Memphis, TN 38152, USA

**Keywords:** Lipolysis, Thermogenesis, Fat mobilization, Weight loss, Nutritional supplements

## Abstract

**Background:**

Higenamine, also known as norcoclaurine, is an herbal constituent thought to act as a beta-2 adrenergic receptor agonist—possibly stimulating lipolysis. It was the purpose of this study to determine the impact of a higenamine-based dietary supplement on plasma free fatty acids and energy expenditure following acute oral ingestion.

**Methods:**

Sixteen healthy subjects (8 men; 26.1 ± 2.5 yrs; 8 women 22.4 ± 3.1 yrs) ingested a dietary supplement containing a combination of higenamine, caffeine (270 mg), and yohimbe bark extract or a placebo, on two separate occasions in a double-blind, randomized, cross-over design, separated by 6–8 days. Blood samples were collected immediately before ingestion, and at 30, 60, 120, and 180 minutes post ingestion, and analyzed for plasma free fatty acids (FFA) and glycerol. Breath samples were collected at the same times for a measure of kilocalorie expenditure and respiratory exchange ratio (RER) using indirect calorimetry. Heart rate and blood pressure were recorded at all times. Data collection occurred in the morning following a 10 hour overnight fast.

**Results:**

A condition effect was noted for both FFA (p < 0.0001) and kilocalorie expenditure (p = 0.001), with values higher for supplement compared to placebo at 60, 120, and 180 minutes post ingestion. No statistically significant effects were noted for glycerol or RER (p > 0.05). A condition effect was noted for heart rate (p = 0.03) and systolic blood pressure (p < 0.0001), with values higher for supplement compared to placebo.

**Conclusion:**

Ingestion of a higenamine-based dietary supplement stimulates lipolysis and energy expenditure, as evidenced by a significant increase in circulating FFA and kilocalorie expenditure. The same supplement results in a moderate increase in heart rate (~3 bpm) and systolic blood pressure (~12 mmHg), which is consistent with previous studies evaluating moderate doses of caffeine and yohimbine, suggesting that higenamine contributes little to the increase in these hemodynamic variables. These findings are in reference to young, healthy and active men and women.

## Background

Obesity has grown to epidemic proportions in recent years, with an estimated one-third of adults in the United States being diagnosed as obese [1]. Ideal treatment for this disease includes appropriate dietary intake [2], coupled with regular physical activity [3] and structured exercise [4]. In some cases, the use of selected dietary supplements may assist in weight lost, as many have shown promise with regards to decreasing appetite [5-7], stimulating lipolysis [8-10], and increasing energy expenditure [8,9,11,12].

Related to the latter, caffeine is likely the most widely used dietary ingredient within weight loss supplements. A naturally occurring crystalline xanthine alkaloid, caffeine is regularly consumed by humans from around the world—being contained within beverages (e.g., coffee, tea, energy drinks) and dietary supplements, as well as medications. In addition to providing a short-term “boost” in energy, caffeinated products have attracted attention due to their potential health-related properties. Particularly, caffeine contributes to improving metabolic capacity and may ameliorate risk factors related to obesity [13] and type-2 diabetes mellitus [14]. While caffeine alone may have beneficial effects and is generally well-tolerated, other ingredients are often looked to in an attempt to enhance the lipolytic and thermic effects of caffeine.

One such ingredient is yohimbe bark extract, an herbal agent found naturally in *Pausinystalia yohimbe* (Yohimbe), *Rauwolfia serpentina* (Indian Snakeroot), and *Alchornea floribunda* (Niando) [15,16]. While yohimbe has been used for many years to treat sexual dysfunction in men, more recently, it has been reported that yohimbine, present within yohimbe bark, promotes an improvement in lipid mobilization and thermogenesis. Specifically, yohimbine has been shown to stimulate lipid mobilization in normal weight [17] and obese [18] individuals. Further, an animal study demonstrated that yohimbine administration increased energy expenditure in dogs, suggesting this agent as a mediator for increased thermogenesis [19].

One additional herbal agent that is beginning to receive attention within the dietary supplement community is higenamine, which is found naturally in a variety of plants including *Nelumbo nucifera* (lotus seeds), *Nandina domestica* (fruit), *Aconitum carmichaelii* (root), *Asarum heterotropioides*, *Galium divaricatum* (stem and vine), and *Annona squamosa*. Higenamine, 1-benzyl-1,2,3,4-tetrahydroisoquinoline alkaloid, has been used as a cardiac stimulant through chronotropic and inotropic action. This plant-based agent has been applied using a pharmacological approach in an attempt to improve cardiac function due to its ability to stimulate beta-adrenergic receptors (β-AR). It has been reported that β-AR agonists enhance lipolysis and thermogenesis [19]. Based on this finding, higenamine, as a potent β-AR agonist, may be considered an effective anti-obesity agent, if capable of increasing both lipolytic and thermogenic processes.

While certain studies have been conducted to determine the potential mechanisms of action of higenamine, we are unaware of any studies involving oral intake of higenamine by human subjects. Considering that this ingredient is now making its way into the dietary supplement marketplace, it is of interest to generate data pertaining to the potential efficacy of this herbal ingredient. Moreover, it is important to understand the impact of this agent on the heart rate and blood pressure response to treatment—as the safety of an herbal preparation should always be considered before use. Therefore, the purpose of the present study was to determine the impact of a higenamine-based dietary supplement on plasma free fatty acids and energy expenditure following acute oral ingestion, while measuring the heart rate and blood pressure response to acute oral treatment.

## Results

### Overview: dietary data and subjective response to supplement and placebo

All 16 subjects successfully completed all aspects of the study. Subject data are provided in Table 1. Dietary data were not different between the 24 hours prior to each condition (p > 0.05). Dietary data are presented in Table 2. Subjects tolerated the supplement and placebo conditions well. As expected, selected subjects reported feeling “stimulated” approximately one hour following ingestion of the supplement. That said, no subject experienced an adverse event, with only moderate increases in both heart rate and blood pressure noted (as indicated below; see also Table 3).

**Table 1:** Characteristics of 8 men and 8 women

**Variable**	**Men**	**Women**
Age (yrs)*	26.1 ± 2.5	22.4 ± 3.1
Height (cm)*	176.1 ± 6.7	165.3 ± 6.1
Weight (kg)*	80.2 ± 11.9	62.0 ± 7.9
BMI (kg∙m^-2^)*	25.8 ± 3.5	22.6 ± 2.2
Waist (cm)*	82.8 ± 7.3	68.5 ± 4.6
Hip (cm)	101.7 ± 4.9	97.6 ± 4.3
Waist:Hip*	0.81 ± 0.05	0.70 ± 0.03
Years anaerobic exercise training	8.6 ± 7.9	3.9 ± 3.3
Hours per week anaerobic exercise	2.7 ± 2.8	1.9 ± 1.2
Years aerobic exercise training	10.0 ± 5.5	8.0 ± 5.4
Hours per week aerobic exercise	4.0 ± 2.9	4.2 ± 2.3

Data are mean ± SD.

*men different than women (p < 0.05).

**Table 2:** Dietary data of 16 subjects during the 24 hour period before ingestion of supplement or placebo

**Variable**	**Supplement**	**Placebo**
Kcal	2202 ± 199	2177 ± 225
Protein (g)	100 ± 13	99 ± 15
Carbohydrate (g)	254 ± 26	251 ± 27
Fat (g)	82 ± 12	83 ± 11
Vitamin C (mg)	135 ± 42	129 ± 34
Vitamin E (mg)	14 ± 3	11 ± 3
Vitamin A (RE)	518 ± 173	320 ± 74

Data are mean ± SEM.

No statistically significant differences noted (p > 0.05).

**Figure 1:**
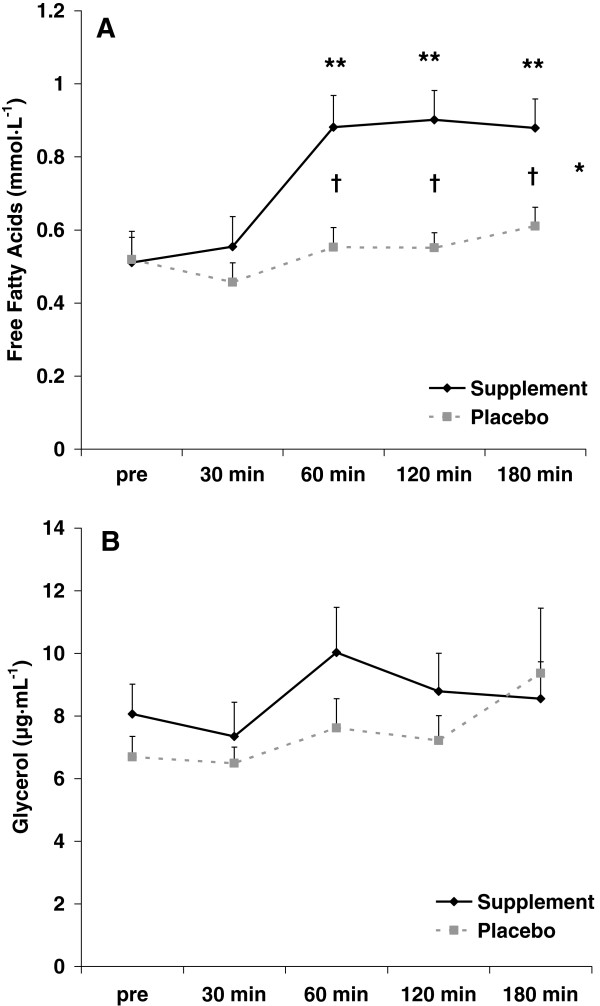
**Plasma free fatty acids (A) and glycerol (B) before and following ingestion of supplement or placebo.** Data are mean ± SEM. *Condition effect noted for free fatty acids (p < 0.0001). **Time effect noted for free fatty acids (p = 0.0009); values higher at 60 min, 120 min, and 180 min compared to 30 min; values higher at 180 min compared to pre. †Difference noted at 60 min (p = 0.0004), 120 min (p = 0.0004), and 180 min (p = 0.004) between supplement and placebo. Interaction effect noted for free fatty acids (p = 0.05). No statistically significant effects noted for glycerol (p > 0.05).

**Table 3:** Heart rate (bpm) and blood pressure (mm Hg) before and following ingestion of supplement or placebo

**Time**	Heart rate Supplement*	**Heart rate *Placebo***	Systolic BP Supplement*	**Systolic BP *Placebo***	**Diastolic BP *Supplement***	**Diastolic BP *Placebo***
Pre	63 ± 3	64 ± 3	112 ± 2	110 ± 2	66 ± 2	64 ± 2
30 min	62 ± 3	62 ± 2	116 ± 3	109 ± 2	68 ± 2	66 ± 2
60 min	65 ± 4	61 ± 2	124 ± 3	106 ± 3	70 ± 2	65 ± 2
120 min	66 ± 4	60 ± 2	122 ± 3	111 ± 2	69 ± 2	66 ± 3
180 min	66 ± 4	60 ± 2	119 ± 3	112 ± 3	67 ± 2	66 ± 3

Data are mean ± SEM.

*Condition effect noted for heart rate (p = 0.03) and systolic blood pressure ( <0.0001). Interaction effect noted for systolic blood pressure (p = 0.03). No other statistically significant effects noted (p > 0.05).

### Biochemical data

Regarding FFA, a condition effect was noted (p < 0.0001), with values higher for the supplement compared to placebo. A time effect was also noted (p = 0.0009), with values higher at 60 minutes, 120 minutes, and 180 minutes compared to 30 minutes; values were also higher at 180 minutes compared to pre. An interaction effect was noted (p = 0.05). Contrasts revealed significant differences between supplement and placebo at 60 minutes (p = 0.0004), 120 minutes (p = 0.0004), and 180 minutes post ingestion (p = 0.004). Regarding glycerol, no condition (p = 0.20), time (p = 0.27), or interaction (p = 0.72) effects were noted. Data for FFA and glycerol are presented in Figure 1. Men and women responded in a similar manner to supplement and placebo with regards to FFA and glycerol.

### Metabolic data

**Figure 2:**
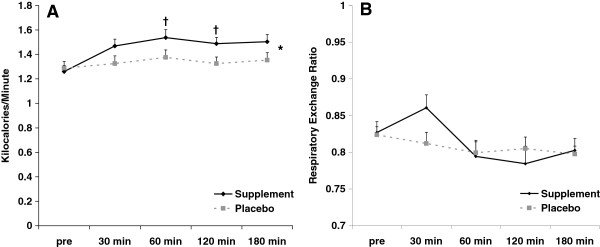
**Kilocalorie expenditure (A) and respiratory exchange ratio (B) before and following ingestion of supplement or placebo.** Data are mean ± SEM. *Condition effect noted for kilocalories (p = 0.001). †Difference noted at 60 min (p = 0.03) and 120 min (p = 0.02) between supplement and placebo; trend for a difference noted at 180 min (p = 0.07). No other statistically significant effects noted for kilocalories or for respiratory exchange ratio (p > 0.05).

Regarding kilocalorie expenditure, a condition effect was noted for kilocalorie expenditure (p = 0.001). No time (p = 0.12) or interaction (p = 0.32) effects were noted for kilocalorie expenditure. Contrasts revealed significant differences between supplement and placebo at 60 minutes (p = 0.03) and 120 minutes (p = 0.02) post ingestion. A trend for a difference was noted at 180 minutes (p = 0.07) post ingestion. Regarding RER, no condition (p = 0.81), time (p = 0.08), or interaction (p = 0.42) effects were noted. Data for kilocalorie expenditure and RER are presented in Figure 2. As expected, energy expenditure for women was lower than for men (sex effect: p < 0.0001), while the RER was not different between men and women (sex effect: p = 0.27).

### Heart rate and blood pressure data

For both heart rate (p = 0.03) and systolic blood pressure (p < 0.0001), a condition effect was noted, with values higher for supplement compared to placebo. No time (p = 0.98) or interaction (p = 0.76) effects were noted for heart rate. No time (p = 0.29) effect was noted for systolic blood pressure; however an interaction effect was noted (p = 0.03). Regarding diastolic blood pressure, no condition (p = 0.11), time (p = 0.90), or interaction (p = 0.88) effects were noted. Data for heart rate and blood pressure are provided in Table 3. The overall heart rate for women was slightly higher than for men (sex effect: p = 0.001), while systolic and diastolic blood pressure was lower (sex effect: p = 0.02 and p = 0.0004, respectively).

## Discussion

The present study documents for the first time the impact of an orally administered higenamine-based dietary supplement on measures of lipolysis and metabolic rate in a sample of human subjects. Our data indicate that when combined with caffeine and yohimbe bark extract, higenamine increases both lipolysis and energy expenditure, as evidenced by a significant increase in circulating FFA and kilocalories. These findings are in reference to young, healthy and active men and women. Future studies may include a sample of older, overweight, and/or inactive individuals to determine if similar results are observed—in particular when considering that such individuals might be consumers of weight loss dietary supplements containing higenamine.

From a mechanistic point of view, caffeine is known to stimulate lipolysis in a variety of ways. One of the plausible mechanisms is that caffeine may reduce the degradation of cyclic adenosine monophosphate (cAMP) and increase cAMP production as well via β-AR independent and dependent pathways [20]. The independent effects may be due to the observation that caffeine appears to antagonize adenosine receptors and also inhibits the activation of phosphodiesterase, which stimulates cAMP degradation [21]. Caffeine also induces an elevation in catecholamine release, which may be secondary to the adenosine blockade [20]. Taken together, caffeine may enhance lipid mobilization, which may have implications for helping to control the onset and progression of obesity.

Although not as well described in the literature, yohimbe bark extract has also been reported to increase lipolysis. Yohimbine, known as an alpha2-adrenoreceptor (α_2_-AR) antagonist, may contribute to enhancing lipid mobilization. Since α_2_-AR functions as an anti-lipolytic mediator, the ability of yohimbine to block α_2_-AR on the fat cells can stimulate fat metabolism. In agreement with this assertion, Galitzky et al. [19] reported greater plasma non-esterified fatty acids and energy expenditure after acute yohimbine ingestion in the dog. This finding agrees with results from human studies which demonstrated that yohimbine administration improved lipolytic capacity by promoting β-AR and inhibiting α_2_-AR in adipocytes in healthy [17] and obese [18] individuals.

Studies using caffeine and yohimbe alone have noted an increase in both markers of lipolysis and metabolic rate. In relation to caffeine use, Rumpler and colleagues [22] found that caffeine treatment (270 mg) enhanced fat oxidation by 8% in men, while increasing energy expenditure by 331 kJ (3.4%). In relation to yohimbe use, yohimbine consumption (0.2 mg∙kg^-1^) increased norepinephrine approximately 40 to 50%, resulting in elevated lipolysis by stimulating β-AR in healthy men [17]. However conflicting data indicate no effect of yohimbine administration on lipolysis [23].

Higenamine has been investigated recently and is beginning to receive attention as a dietary ingredient for inclusion within weight loss supplements. Higenamine has been used to improve cardiovascular and respiratory disease due to its ability as a β-AR agonist. However, to our knowledge, no study has reported pro-lipolytic and/or -thermogenic properties of higenamine alone or combined with other ingredients in humans. We observed a significant increase in lipolysis and thermogenesis, noted as greater plasma FFA and energy expenditure in the supplement group compared with the placebo group. As previously mentioned, β-AR agonists enhance lipolysis and thermogenesis by stimulating related signaling pathways.

While caffeine can increase metabolic rate and may have lipolytic potential, some studies indicate that caffeine does not contribute to lipolysis. For example, Bracco et al. [24] reported that while caffeine (1248 mg∙d^-1^ for lean; 1604 mg∙d^-1^ for obese) increased energy expenditure by 728 kJ (7.6%) and 410 kJ (4.9%) in lean and obese women, respectively over 24 hours, it did not increase fat oxidation in either group. On the other hand, it has been suggested that caffeine administration combined with other ingredients may enhance both energy expenditure and lipolysis. Rumpler and colleagues [22] reported that adult men who consumed caffeine (270 mg) along with catechins (662.5 mg) significantly increased both energy expenditure (2.9%) and fat oxidation (12%) over 24 hours, compared with 3.4% and 8% for caffeine alone. These findings agree with more recent work by Rudelle et al. [25] who observed that combined administration of caffeine (300 mg) and catechins (540 mg) improved metabolic capacity (4.6%) and lipolysis (3.5%) in men and women. Further, Dulloo et al. [26] reported that 150 mg of caffeine ingestion alone did not increase energy expenditure and lipid mobilization, while caffeine combined with catechin polyphenols increased energy expenditure by 328 kJ (4%) and fat oxidation by 9.9%.

Considering the reported isolated impact of caffeine and yohimbe on markers of lipolysis and metabolic rate (as described above), the inclusion of higenamine in the tested supplement likely had an impact on both FFA and kilocalorie expenditure. Of course, future studies should be designed to deliver each of these three agents independently, in order to better understand their isolated impact on selected measures of lipolysis and metabolic rate. Our failure to include all three ingredients independently is a limitation of the present design.

Based on our findings for increased lipolysis and kilocalorie expenditure, it might be hypothesized that the supplement may aid in body weight/fat loss over time. When considering the energy expenditure data, the supplement resulted in an approximate increase of 10 kilocalories per hour over placebo during the post ingestion observation period. If this increase persisted, the increased energy expenditure may translate into meaningful weight loss over time. It is interesting to note that values for kilocalorie expenditure were highest at the 180 minute post ingestion time, suggesting that the increase may have persisted at times beyond this point. Our cessation of measurements at 180 minutes post ingestion may be considered a limitation of the present design. Further research is needed to determine the impact of this supplement on weight/fat loss when used on a regular basis—possibly in the context of an acute exercise session, as ingesting the supplement prior to exercise might make available more FFAs for oxidation during the actual exercise session.

If considering long-term treatment with this supplement, it is important to take into account the rise in heart rate and blood pressure experienced by subjects. As indicated in Table 3, heart rate and blood pressure (systolic in particular) were moderately elevated following ingestion of the supplement. When compared to use of caffeine alone (at a dosage of ~250-300 mg; a similar amount contained within 2–3 cups of coffee), the changes observed with the supplement are slightly greater. Corti et al. [27] found that intravenous caffeine administration (250 mg) significantly increased systolic blood pressure by 3 mmHg and 6.4 mmHg at 30 and 60 minutes. Lane [28] also reported that caffeine consumption (250 mg) significantly increased both systolic and diastolic blood pressure approximately 7 mmHg and 6 mmHg, respectively. Hartley and colleagues [29] reported that caffeine consumption (3.3 mg·kg^-1^) increased both systolic and diastolic blood pressure by 4.5 and 3.3 mmHg in women and by 4.1 and 3.8 mmHg in men. Similar findings for blood pressure have been observed with yohimbine administration, with an increase in mean arterial pressure of 13% [30] and 16% [31].

Regarding heart rate change, caffeine administration most often results in either no change or a slight decrease in resting heart rate. However, yohimbine administration has been noted to induce a heart rate increase of 8% in healthy individuals [31]. Collectively considering the above, our observed increase of ~3 bpm in heart rate and ~12 mmHg in systolic blood pressure may be mostly explained by the combined administration of caffeine and yohimbe bark extract. Interestingly, higenamine administration has been reported to actually decrease blood pressure in animals [32], and was concluded to be safe for human intake following acute intravenous administration [33]. This is supported by evidence indicating a relatively high lethal dose (LD_50_) of 50 mg/kg following higenamine injection, suggesting less concern for acute toxicity with oral consumption [34].

With consideration for the above information, future studies should ideally include the administration of higenamine alone to determine the influence of this agent on both heart rate and blood pressure. In fact, future work using a wide variety of subjects, coupled with a broad array of outcome measures would be helpful in determining the overall safety profile of higenamine and higenamine-based dietary supplements. For now, it would be wise for individuals who are hypertensive (resting blood pressure ≥140/90 mmHg) or for those who are pre-hypertensive (resting blood pressure ≥120/80 mmHg) to avoid using any stimulant-based dietary supplements. As with the use of any weight/fat loss aid, individuals should consult with a qualified health care professional prior to use.

## Conclusions

We report that a higenamine-based dietary supplement stimulates lipolysis and energy expenditure in young and healthy human subjects. The same supplement results in a moderate increase in heart rate (~3 bpm) and systolic blood pressure (~12 mmHg). Because the supplement contained a combination of higenamine, caffeine, and yohimbe bark extract, we are uncertain of the exact contribution of each of the three ingredients. Further well-controlled intervention trials are needed to determine the chronic effects of the supplement on body weight/fat loss and associated health related parameters.

## Methods

### Subjects

Healthy, exercise-trained men (n = 8) and women (n = 8) were enrolled in this study and completed all aspects of this work. Prior to participation, subjects completed a health history and physical activity questionnaire. Subjects did not have any history of cardiovascular or metabolic disease, nor did any subject smoke cigarettes. All subjects were physically active, engaged in regular anaerobic and/or aerobic exercise. Descriptive characteristics of subjects are presented in Table 1. The study protocol was approved by The University of Memphis Institutional Review Board for Human Subjects Research and subjects provided written informed consent prior to participating.

### Conditions and lab testing

Following the initial screening procedures, subjects reported to the lab for testing in the morning hours (0600–1000), following a 10 hour overnight fast and without caffeine for the prior 24 hours. Testing was conducted on two different occasions separated by 6–8 days. The time of day for testing was matched for each subject on the two occasions. All procedures, as described below, were identical for both test sessions (supplement and placebo). The supplement consisted of a proprietary combination of higenamine, yohimbe bark extract, and caffeine (270 mg). The total dosage of each ingredient was delivered by ingesting two caplets. The placebo caplets contained microcrystalline cellulose; subjects also ingested two caplets of the placebo. For each condition, caplets were dispensed from the same bottle and were produced in accordance with Good Manufacturing Practices. Caplets for both conditions were identical in appearance and the experiment was conducted as a double blind, randomized, cross-over design. The investigators did not receive the blinding code until all data were collected. No food was allowed during the three hour post ingestion period. However, water was allowed *ad libitum* and was measured and matched for both days of testing (mean intake for men = 1272 ± 124 mL; mean intake for women = 760 ± 117 mL).

Subjects were asked not to exercise or to perform any strenuous physical activity for the 48 hours prior to each test day. Following a minimum10 minute period of quiet rest, heart rate (via 60 second radial artery palpation) and blood pressure (via auscultation) were measured, a blood sample was obtained, and subjects provided a five-minute breath sample (for analysis of kilocalorie expenditure and respiratory exchange ratio [RER]). Subjects were then provided with their assigned condition and ingested it in the presence of an investigator. At all other measurement times (30, 60, 120, and 180 minutes post ingestion), the same order of collection as described above was followed. Subjects remained inactive in the laboratory during the entire three hour test period and read, listened to music, watched television, worked on a computer, etc.

A total of five venous blood samples (~7 mL per sample) were taken from subjects’ forearm vein via needle and collection tube (pre ingestion, 30, 60, 120, and 180 minutes post ingestion). Blood was immediately processed in a refrigerated centrifuge in order to obtain plasma. The plasma samples were then stored in aliquots at −70°C. Assays were performed in duplicate on first thaw within one month of sample collection. Free fatty acids were determined using a fatty acid detection kit (Catalogue # SFA-5; Zen-Bio, Inc.; Research Triangle Park, NC) following the instructions of the manufacturer. Glycerol was determined using the Free Glycerol Determination Kit (FG0100) and Glycerol Standard (G7793) following the instructions of the manufacturer (Sigma Aldrich; St. Louis, MO).

The measurement of kilocalorie expenditure was performed using indirect calorimetry (Parvo Medics, TrueOne® 2400). All equipment was calibrated on the morning of each test day. Total oxygen consumption (L∙min^-1^) was determined from gas collection and used to estimate total kilocalorie expenditure. The RER was also determined from gas collection data (VCO_2_/VO_2_), and used as a measure of substrate utilization.

### Dietary intake

Subjects were asked to record all food and beverage consumed during the 24 hour period prior to each test day. Subjects were asked to duplicate the food and beverage intake during the 24 hour periods prior to both test days, in an attempt to best control for the influence of acute dietary intake on our outcome measures. Diet records were analyzed for total kilocalories, protein, carbohydrate, fat, and selected vitamins (Food Processor SQL, version 9.9, ESHA Research, Salem, OR).

### Statistical analysis

Data were analyzed using a 2 (condition) by 5 (time) analysis of variance (ANOVA). Tukey’s *post hoc* testing was used when needed. Single degree of freedom contrasts were used to investigate differences in FFA and kilocalorie expenditure between supplement and placebo at the post ingestion time points. Dietary and subject descriptive data were analyzed using a one-way ANOVA. All analyses were performed using JMP statistical software (version 4.0.3, SAS Institute, Cary, NC). Statistical significance was set at P ≤ 0.05. The data are presented as mean ± SEM, except for subject descriptive characteristics (mean ± SD). Although a comparison between men and women was not a primary focus of this study, we did conduct an analysis in which sex was built into the model. These results are very briefly presented within the Results section.

## Competing interests

Financial support for this work was provided in part by USPlabs, LLC. None of the authors have a financial interest in this company. RJB has received research funding or acted as consultant to other nutraceutical and dietary supplement companies. All other authors declare no competing interests.

## Authors’ contributions

SRL, JMS, TAG, and ICH were responsible for subject recruitment, data collection, blood collection and processing, data entry, and assistance with manuscript preparation. RJB was responsible for the study design, biochemical work (with assistance of SRL), statistical analyses, and manuscript preparation. All authors read and approved of the final manuscript.
